# Incidence, Characteristics, and Management Pathways of Obstructive Sleep Apnea in England: A 10-Year Retrospective Cohort Study

**DOI:** 10.1016/j.chpulm.2025.100221

**Published:** 2025-10-10

**Authors:** Asad Ali, Melike Deger, Ankit Ghildiyal, Naomi Alpert, Fatemeh Saberi Hosnijeh, Stephen Boult

**Affiliations:** a University Hospital Coventry & Warwickshire NHS Trust, Coventry, England; b ResMed Science Center, ResMed, Martinsried/Didcot/San Diego, Germany/UK/USA; c OPEN Health, Rotterdam, The Netherlands; d OPEN Health, London, England

**Keywords:** burden of disease, CPAP, facility and services utilization, OSA, quality of health care

## Abstract

**Background:**

OSA is common and can be effectively treated with CPAP. Up-to-date English epidemiologic data to inform care are scarce.

**Research Question:**

What are the incidence of OSA, patient characteristics, comorbidities, diagnostic methods, treatment pathways, and waiting times in England?

**Study Design and Methods:**

This retrospective cohort study used the Clinical Practice Research Datalink and linked Hospital Episode Statistics databases from January 1, 2010, to December 30, 2020. Included adults had ≥ 1 OSA code and ≥ 12 months’ medical records before/after OSA diagnosis. Participant subgroups were defined by age and diagnosis/treatment (OSA + CPAP, OSA without CPAP, and no OSA). The OSA incidence was determined, and baseline sociodemographic characteristics/comorbidities, diagnosis/treatment pathways, and patient waiting times were recorded. The primary analysis population included data from 2010 to 2019 (to exclude the health effects of the COVID-19 pandemic); sensitivity analysis determined the OSA incidence rate for the full data set.

**Results:**

The primary analysis population included 69,155 individuals (nearly 70% male; mean age, 53.4 ± 13.2 years; mean BMI, 36.0 ± 8.5 kg/m^2^; median follow-up, 45 months); there were 13,058, 16,068, and 40,029 in the OSA + CPAP, OSA without CPAP, and no OSA groups, respectively. Obesity and cardiometabolic disorders were the most common comorbidities. The incidence of OSA increased from 46.74 per 100,000 in 2010 to 91.04 per 100,000 in 2019, with an average of 65.06 per 100,000 person-years over the period. Median waiting times were 37 days from general practitioner referral to sleep clinic visit, 50 days from sleep clinic visit to OSA diagnosis, 70 days from diagnosis to CPAP initiation, and 194 days from general practitioner referral to CPAP initiation. These medians represent independent intervals and may not sum to the total journey time. Forty-two percent of patients had no follow-up sleep clinic visits after starting CPAP.

**Interpretation:**

These data highlight the increasing burden of OSA in England and underscore the need for improved diagnostic/treatment frameworks to expedite OSA management.

**Take-Home Points**

**Study Question:** How has the burden of OSA changed in England over the past decade, and what are the trends in diagnosis, treatment pathways, and follow-up care?

**Results:** The incidence of OSA nearly doubled from 2010 to 2019; however, care delays remain substantial—median time from general practitioner referral to CPAP initiation was > 6 months, and 42% of treated patients received no follow-up sleep clinic visits.

**Interpretation:** These findings underscore the growing impact of OSA in England and highlight urgent gaps in care delivery, calling for more efficient, guideline-driven diagnostic and treatment strategies to improve outcomes

Characterized by repetitive episodes of upper airway collapse during sleep, OSA is a common chronic condition in adults.^1^, ^2^, ^3^ In the United Kingdom, 5% of people between 30 and 70 years of age (30 million out of a total population of 66 million) have moderate or severe OSA (1.5 million people), and an additional 20% have mild OSA; thus, an estimated 25% of the UK population (8 million people) have mild, moderate, or severe OSA.^1^ Risk factors for OSA include male sex, older age, and obesity.^4^, ^5^, ^6^ Therefore, the aging global population and the rising prevalence of obesity are important contributors to increasing OSA rates. In England, 2 of every 3 adults are overweight or obese,^7^ and the rate of sleep apnea has increased significantly over the last 20 years.^8^

Randomized controlled trials have established the efficacy and safety of CPAP therapy for controlling abnormal respiratory events during sleep, reducing daytime sleepiness and improving daytime functioning in people with OSA.^9^^,^^10^ In the United Kingdom, National Institute for Health and Care Excellence (NICE) guidelines for OSA/hypopnea syndrome in people > 16 years of age recommend that CPAP therapy be prescribed for individuals with mild OSA who have symptoms that affect their quality of life and usual daily activities, and for individuals with moderate or severe OSA.^11^ It has been estimated that there are 1.75 million people with moderate-to-severe OSA in the United Kingdom who could benefit from CPAP therapy.^12^ However, most of these individuals remain untreated.^12^

Underdiagnosis and undertreatment are important issues in OSA management, with approximately 80% of people who have OSA remaining undiagnosed, and therefore untreated.^13^, ^14^, ^15^ The implications of undiagnosed and untreated OSA include increased risk of cardiometabolic diseases and stroke, excessive daytime sleepiness, workplace errors or accidents, road traffic accidents, reduced quality of life, increased health care resource utilization, and death.^16^, ^17^, ^18^ Rates of multimorbidity in people with undiagnosed OSA are high.^17^

When managing patients with OSA being treated with CPAP, the NICE guidelines^11^ recommend the use of telemonitoring with face-to-face, video, or phone consultations (including review of telemonitoring data). Specifically, the first consultation should take place within 1 month after CPAP therapy initiation with subsequent follow-up to achieve optimal control of the apnea-hypopnea index, oxygen desaturation index, and symptoms. Once CPAP has been optimized, annual follow-up is recommended.

Epidemiologic studies of individuals with OSA have been conducted in Europe to facilitate understanding of the size and characteristics of this population, and the nature and delivery of treatment.^13^^,^^19^ Currently available data for the United Kingdom^1^ only relate to patients with obesity.^20^ Therefore, this study was designed to provide current epidemiologic evidence regarding the burden of OSA in England, along with patient characteristics, comorbidities, treatment pathways, and patient journey times.

## Study Design and Methods

### Study Design and Data Source

This retrospective cohort study used the Clinical Practice Research Datalink (CPRD) Gold and Aurum databases, primary care databases that cover approximately 20% of the UK population^21^^,^^22^ and are linked at the patient level to the Hospital Episode Statistics (HES) database.^23^ CPRD was also linked to the Office for National Statistics death registry^24^ and patient-level Index of Multiple Deprivation data via the CPRD.^25^ The study observation period was from January 1, 2009, to March 21, 2021, and the participant eligibility period was January 1, 2010, to March 31, 2020. Data were obtained by application to the CPRD, and the study protocol was approved by the Research Data Governance committee (No. 22_002154), as per the required process.^26^

### Diagnoses and Index Date

The index date was defined as the date of first diagnosis of OSA in the CPRD during the participant eligibility period. Data on the following patient characteristics were obtained: age at OSA diagnosis, biological sex, Index of Multiple Deprivation quintile, smoking status, body weight, BMI, the top 10 most frequent baseline diagnoses in CPRD-HES linked data, and OSA-related comorbidities in the preindex period (the time from the patient’s registration in the CPRD or January 1, 2009, whichever was earlier, to the index date [exclusive]). Evaluations of interest were pulse oximetry, home sleep study, and full polysomnography (PSG), plus tools commonly used for initial screening and risk stratification such as the Epworth Sleepiness Scale (ESS) and the Berlin Questionnaire. Positive airway pressure with CPAP or automatically titrating CPAP was recorded (hereafter referred to as CPAP). In addition, the number of sleep clinic visits per year after CPAP initiation was recorded. A full list of diagnostic codes for OSA (e-Table 1), positive airway pressure therapy (e-Table 2), and diagnostic tests (e-Tables 3, 4) are provided.

### Participants

All eligible patients were included during the eligibility period. Eligible patients were permanently registered as acceptable individuals in the CPRD-HES linked database (acceptable is a flag in the CPRD database that signifies that an individual’s records are of sufficient quality to be used in research studies), ≥ 18 years of age, and had a first recorded diagnosis of OSA in the eligibility period (January 1, 2010-March 31, 2020). Individuals with < 12 months of baseline data before the index date or < 12 months of follow-up data after the index date, those who used bilevel positive airway pressure therapy at any time during follow-up, people with a diagnosis of central sleep apnea or nocturnal hypoventilation after the index OSA diagnosis, individuals who underwent tonsillectomy, adenoidectomy, or uvulopalatopharyngoplasty after the index date, and pregnant women were excluded.

### Patient Subgroups

Patients were divided into 3 groups based on diagnosis/treatment: (1) confirmed OSA diagnosis based on the presence of 2 OSA codes then treatment with CPAP/automatically titrating CPAP (OSA + CPAP group); (2) confirmed OSA diagnosis based on the presence of 2 OSA codes but not treated with CPAP/automatically titrating CPAP (OSA without CPAP group); and (3) people with only 1 OSA code who may have had suspected OSA but did not have a confirmed diagnosis and had no further record of OSA coded in records, either because they did not have OSA or did not go to the sleep clinic (no OSA group).

### Objectives

The primary objectives were as follows: (1) to determine the diagnosed incidence of OSA; (2) to describe the baseline sociodemographic characteristics and comorbidities of the study population, overall and in subgroups based on diagnosis/treatment; (3) to describe treatment pathways in newly diagnosed individuals with OSA (overall and broken down by region); and (4) to describe the annual frequency of sleep clinic visits after CPAP initiation.

Additional exploratory objectives were to describe waiting times throughout the OSA diagnosis and treatment pathway, overall and in groups based on diagnosis/treatment.

### Statistical Analyses

Data are presented using descriptive statistics: frequency and percentages for categorical variables and mean ± SD or median (interquartile range [IQR]) as appropriate for continuous variables. Missing data were not substituted or imputed.

The incidence of OSA was estimated by dividing the number of new OSA diagnoses in the observation period by the number of person-years at risk. The incidence was reported overall and for each calendar year in the observation period; 95% CI values were estimated assuming a Poisson distribution.

To exclude the health effects of the COVID-19 pandemic, the primary analysis population included all patients recruited up to December 31, 2019 (ie, database cutoff date: December 31, 2019). A sensitivity analysis repeated all analyses for the full set of included patients (primary analysis population plus those enrolled from January 1, 2020, to December 31, 2020; sensitivity analysis population).

## Results

### Study Population

The primary analysis population included 69,155 individuals, including 13,058 in the OSA + CPAP group, 16,068 in the OSA without CPAP group, and 40,029 in the no OSA group. Most patients (nearly 70%) were male, the mean age ± SD at diagnosis was 53.4 ± 13.2 years, and the mean BMI ± SD was 36.0 ± 8.5 kg/m^2^. Sex, mean age, and age distribution were similar across the 3 groups (Table 1). After obesity, the most common comorbidities across all 3 subgroups were cardiovascular disease, metabolic disorders, respiratory disorders, and psychiatric disorders (Table 1).

**Table 1:** Participant Characteristics in the Overall Study Population and by Diagnosis/Treatment Subgroup

Characteristic	Overall (N = 69,155)	OSA + CPAP (n = 13,058)	OSA Without CPAP (n = 16,068)	No OSA (n = 40,029)
Age at diagnosis, y	53.4 [13.2]	53.8 [12.7]	53.0 [13.1]	53.5 [13.4]
Age group, y				
18-20	245 (0.35)	30 (0.23)	55 (0.34)	160 (0.40)
21-30	2,920 (4.22)	440 (3.37)	695 (4.33)	1,780 (4.45)
31-40	8,670 (12.54)	1,545 (11.83)	2,080 (12.94)	5,040 (12.59)
41-50	16,620 (24.03)	3,145 (24.08)	3,985 (24.80)	9,490 (23.71)
51-60	19,655 (28.42)	3,900 (29.87)	4,520 (28.13)	11,230 (28.05)
61-70	13,875 (20.06)	2,705 (20.72)	3,190 (19.85)	7,985 (19.95)
71-80	6,060 (8.76)	1,120 (8.58)	1,305 (8.12)	3,635 (9.08)
81-90	1,080 (1.56)	175 (1.34)	230 (1.43)	675 (1.69)
91-100	35 (0.05)	< 7^a^	< 7^a^	30 (0.07)
Male sex^b^	48,326 (69.88)	9,170 (70.23)	11,436 (71.17)	27,720 (69.25)
Body weight, kg	106.1 [25.4]	107.7 [25.3]	106.5 [25.5]	105.4 [25.4]
Missing	17,619 (25.48)	2,962 (22.68)	4,323 (26.90)	10,334 (25.82)
Body mass index, kg/m^2^	36.0 [8.5]	36.5 [8.5]	36.0 [8.6]	35.8 [8.5]
Missing	17,619 (25.48)	2,962 (22.68)	4,323 (26.90)	10,334 (25.82)
Does not smoke	51,156 (73.97)	9,753 (74.69)	11,678 (72.68)	29,725 (74.26)
Smokes	11,786 (17.04)	2,200 (16.85)	2,767 (17.22)	6,819 (17.04)
Missing	6,213 (8.98)	1,105 (8.46)	1,623 (10.10)	3,485 (8.71)
IMD score (in quintiles)^c^				
≥ 34.18	15,480 (22.38)	3,050 (23.36)	3,665 (22.81)	8,765 (21.90)
21.36-34.17	14,925 (21.58)	2,680 (20.52)	3,400 (21.16)	8,850 (22.11)
13.8-21.35	13,480 (19.49)	2,535 (19.41)	3,130 (19.48)	7,810 (19.51)
8.50-13.79	13,095 (18.94)	2,415 (18.49)	3,120 (19.42)	7,555 (18.87)
≤ 8.49	12,110 (17.51)	2,370 (18.15)	2,735 (17.02)	7,000 (17.49)
Unknown	75 (0.11)	10 (0.08)	15 (0.09)	45 (0.11)
CCI	1.1 [1.5]	1.2 [1.5]	1.1 [1.4]	1.1 [1.5]
CCI group				
0	32,189 (46.55)	5,860 (44.88)	7,683 (47.82)	18,551 (46.34)
1-2 (mild)	26,904 (38.90)	5,184 (39.70)	6,190 (38.52)	15,611 (39.00)
3-4 (moderate)	7,640 (11.05)	1,522 (11.66)	1,674 (10.42)	4,455 (11.13)
5 (severe)	2,422 (3.50)	492 (3.77)	521 (3.24)	1,412 (3.53)
Common comorbidities				
Obesity	57,458 (83.09)	11,257 (86.21)	13,401 (83.40)	32,800 (81.94)
Cardiovascular disorders	26,500 (38.32)	5,425 (41.55)	6,152 (38.29)	14,923 (37.28)
Metabolic disorders	16,582 (23.98)	3,360 (25.73)	3,823 (23.79)	9,399 (23.48)
Respiratory disorders	15,087 (21.82)	2,880 (22.06)	3,473 (21.61)	8,734 (21.82)
Psychiatric disorders	11,025 (15.94)	2,093 (16.03)	2,450 (15.25)	6,482 (16.19)

Data are presented as mean [SD] or No. (%). CCI = Charlson Comorbidity Index; IMD = Index of Multiple Deprivation.

a Small number suppression applied as per best practice guidelines for values < 7.

b Sex was missing for 1 patient.

c Higher scores indicate greater deprivation.

The median follow-up duration was 45 months (IQR, 21-77) overall, 46 months (IQR, 23-77) in the OSA + CPAP group, 54 months (IQR, 29-84) in the OSA without CPAP group, and 40 months (IQR, 18-73) in the no OSA group.

### OSA Incidence Rate

The overall diagnosed incidence of OSA was 65.06 per 100,000 person-years (95% CI, 64.57-65.54). The OSA incidence rate increased steadily over time between 2010 and 2019 (Table 2).

**Table 2:** Incidence Rate of OSA Over Time

Year	OSA Incidence Rate Per 100,000 Person-Years (95% CI)
2010	46.74 (45.46-48.07)
2011	47.97 (46.67-49.31)
2012	50.23 (48.90-51.59)
2013	53.32 (51.94-54.73)
2014	57.38 (55.95-58.85)
2015	65.60 (64.07-67.17)
2016	73.47 (71.86-75.13)
2017	79.77 (78.10-81.48)
2018	83.23 (81.53-84.96)
2019	91.04 (89.27-92.84)
2020	57.84 (65.42-59.29)

### Tests and Assessments

PSG was the most frequently used diagnostic test overall and in the different subgroups. Although not diagnostic for OSA, the ESS was used in a similar proportion of individuals to PSG (Table 3). Despite home respiratory polygraphy being the recommended primary diagnostic test, this was only used in 9% of the study population overall, and use of pulse oximetry was equally low (Table 3).

**Table 3:** Tests and Evaluations in the Overall Study Population and in Subgroups Based on Diagnosis/Treatment

Variable^a^	Overall (N = 69,155)	OSA + CPAP (n = 13,058)	OSA Without CPAP (n = 16,068)	No OSA (n = 40,029)
Berlin questionnaire	179 (0.26)	50 (0.38)	38 (0.24)	91 (0.23)
Epworth Sleepiness Scale	19,077 (27.59)	4,461 (34.16)	4,382 (27.27)	10,234 (25.57)
Home sleep study	6,550 (9.47)	1,470 (11.26)	1,448 (9.01)	3,632 (9.07)
Pulse oximetry	7,716 (11.16)	1,300 (9.96)	1,489 (9.27)	4,927 (12.31)
PSG	19,146 (27.69)	3,833 (29.35)	3,915 (24.37)	11,398 (28.47)
Pulse oximetry only	6,034 (8.73)	1,023 (7.83)	1,183 (7.36)	3,828 (9.56)
Home sleep study only	< 7^b^	< 7^b^	< 7^b^	< 7^b^
PSG only	11,201 (16.20)	2,143 (16.41)	2,224 (13.84)	6,834 (17.07)
Pulse oximetry + home sleep study	< 7^b^	0 (0.00)	< 7^b^	< 7^b^
Pulse oximetry + home sleep study + PSG	276 (0.40)	55 (0.42)	58 (0.36)	163 (0.41)
Home sleep study + PSG	6,265 (9.06)	1,413 (10.82)	1,386 (8.63)	3,466 (8.66)
Pulse oximetry + PSG	1,404 (2.03)	222 (1.70)	247 (1.54)	935 (2.34)

Data are presented as No. (%). PSG = polysomnography.

a Categories are not mutually exclusive and therefore percentages do not sum to 100.

b Due to low number suppression, Clinical Practice Research Datalink-Hospital Episode Statistics database users are not allowed to publish low numbers; this means that any number < 7 is listed as < 7, meaning that the actual number could be anywhere between 1 and 6 (zero is shown as 0).

### Waiting Times

Waiting time data are summarized in Table 4. The overall median waiting time between general practitioner (GP) referral to the first sleep clinic visit was > 1 month (37 days) and, based on the IQR values, 50% of waiting times were between 19 and 63 days; there was variation in waiting times between regions, with a few having much shorter waiting times (close to 0). The median time from the first sleep clinic appointment to OSA diagnosis (first OSA code) was closer to 2 months. The median time from an OSA diagnosis (first OSA code) to CPAP therapy initiation (first CPAP code) was > 2 months, with some regional variations. The total median waiting time between GP referral and CPAP therapy initiation was > 6 months, indicating significant overall delays, and there was considerable variation depending on the region.

**Table 4:** Waiting Times in the Overall Study Population and by Region

Variable	GP Referral to First Sleep Clinic Appointment, d	First Sleep Clinic Appointment to OSA Diagnosis, d	OSA Diagnosis to CPAP Therapy, d	Total Time From GP Referral to CPAP Initiation, d
Overall (N = 69,155)	37 (19-63)	50 (10-147)	70 (0-469)	194 (90-833)
By region (n = 55,530)				
East Midlands (n = 1,690)	35 (22-49)	40 (7-114)	38 (0-212)	122 (70-406)
East of England (n = 3,605)	36 (21-58)	49 (18-148)	46 (0-268)	172 (101-610)
London (n = 1,180)	43 (22-73)	77 (23-286)	77 (0-324)	211 (122-625)
North East (n = 2,735)	10 (0-22)	46 (21-125)	22 (0-212)	121 (67-936)
North West (n = 10,715)	36 (20-58)	48 (11-148)	41 (0-164)	114 (64-377)
South East (n = 14,455)	39 (16-67)	53 (13-109)	26 (0-187)	133 (73-414)
South West (n = 9,325)	42 (27-66)	15 (2-100)	84 (0-531)	260 (87-914)
West Midlands (n = 11,825)	28 (13-48)	72 (28-235)	46 (0-242)	189 (94-634)

Data are presented as No. (%) or median wait times (interquartile range). GP = general practitioner.

### Sleep Clinic Visits During Follow-Up

During the follow-up period, 1,705 of 13,058 CPAP users (13.06%) averaged 0.5 to 1 sleep clinic visit per year, 2,223 (17.02%) averaged 1 to 2 visits per year, and 1,675 (12.83%) averaged > 3 visits per year; 5,463 CPAP users (41.84%) had no sleep clinic visits after CPAP therapy initiation.

### Sensitivity Analysis

The sensitivity analysis population included 75,383 individuals (mean age ± SD, 53.5 ± 13.3 years; nearly 70% male), including 15,616 with OSA + CPAP, 17,257 in the OSA without CPAP group, and 42,510 in the no OSA group; median follow-up was 62 months (IQR, 37-96). Other characteristics (e-Table 5) were consistent with those of the primary analysis population. The overall diagnosed incidence rate of OSA was 64.36 per 100,000 person-years. The incidence rate in 2020 was 57.84 per 100,000 person-years (95% CI, 56.42-59.29) (Table 2), which was lower than any year from 2015 onward. Distribution of tests/evaluations (e-Table 6) and waiting times (e-Table 7), overall and by diagnosis/treatment group, were generally consistent with those seen in the primary analysis population.

## Discussion

Our data showed that the OSA incidence rate increased steadily between 2010 and 2019 (reaching 91.04 per 100,000 person-years). This would equate to 55,534 people diagnosed in 2023 (based on an England population of 61 million). Previous UK data from NHS administrative areas identified regions of high OSA prevalence based on common risk factors but lacked recent incidence estimates.^27^ However, these data are now > 10 years old, and there was no overall incidence/prevalence estimate. Another previous UK study using the CPRD reported an OSA prevalence rate of 5.4% in individuals with obesity.^20^ We report general English OSA incidence data for the first time and provide an up-to-date picture of the size and nature of the OSA burden, and issues relating to OSA diagnosis and treatment.

The rise in OSA incidence is similar to that documented in other epidemiologic studies.^28^ There are many potential explanations for this, especially rising rates of obesity.^29^^,^^30^ In addition, the criteria used to score sleep studies have changed over time, such as the American Academy of Sleep Medicine criteria for scoring hypopneas that changed from a 4% to 3% oxygen desaturation in 2012.^31^ This change was associated with an increase in the prevalence of OSA based on analysis of polysomnographic data.^32^ Another potential contributor to higher OSA incidence is increasing awareness and knowledge of OSA among health care professionals. The retrospective nature of our data analysis means that the contribution of these potential confounders to our findings is unknown.

Another interesting finding of our study was that 58% of individuals had a single OSA record (classified as no OSA), potentially due to unconfirmed diagnoses, missed clinic visits, or coding errors. Our approach to defining patients with OSA is therefore conservative, potentially underestimating the true prevalence of OSA. In addition, some of those included in the no OSA group could have had undiagnosed OSA or been lost to follow-up, emphasizing the need for improved diagnostic and care continuity.

Of the significant proportion of diagnosed OSA cases remaining untreated, many were likely mild OSA managed with mandibular devices or lifestyle interventions because CPAP was not previously recommended for mild OSA. The lack of OSA severity data makes it difficult to determine undertreatment rates, but some untreated patients were likely to be candidates for CPAP. In addition, there are difficulties with mandibular devices regarding adherence tracking and ensuring sustained effectiveness, both of which are critical for managing OSA. In addition, adherence reporting is mandatory for some professions (eg, professional drivers^33^). In 2021, the NICE guidelines were updated to recommend CPAP therapy for mild OSA, underscoring the need to revisit treatment pathways and expand access to effective therapies. The recommendation for CPAP in people with mild OSA, plus advancements such as telemonitoring, should improve adherence and treatment outcomes.

We analyzed data from 2010 to 2019 to avoid the health care impact of the COVID-19 pandemic. The OSA incidence rate in 2020 (57.84 per 100,000) was lower than 2015 to 2019 (65.60-91.04 per 100,000), likely due to reduced clinic visits during the pandemic. There is therefore the potential for an even larger proportion of people with OSA to be undiagnosed and therefore not receiving appropriate treatment designed to prevent the negative health consequences of OSA. Telemedicine and telemonitoring strategies can attenuate or prevent delays to diagnosis and therapy initiation for patients with OSA, and these were put to good use during the COVID-19 pandemic.^34^^,^^35^

The obesity rate in this population was 83%—double that of German (40.5%)^36^ and French (46.2%)^13^ cohorts—suggesting referral bias favoring individuals with obesity. Referral bias could contribute to the underdiagnosis (and therefore undertreatment) of OSA in nonobese and/or asymptomatic individuals. The lack of sleep study referral for nonobese individuals is likely to be a major limitation of OSA services in England. In addition to obesity, our study population often also had cardiometabolic comorbidities, which is consistent with reported associations between OSA and a variety of cardiovascular, cerebrovascular, and metabolic diseases.^37^, ^38^, ^39^, ^40^, ^41^ Interestingly, rates of obesity and other comorbidities were relatively similar in the OSA + CPAP, OSA without CPAP, and no OSA groups. This was somewhat unexpected given the strong association between obesity and OSA. One possibility is that consistent rates of comorbidities across all analyzed groups reflected the data source, with participants having records in both primary care (CPRD) and at the hospital level (HES). The requirement for all participants to have CPRD-HES linked data means that all also interacted with the hospital system and this could be indicative of worse overall health.

Despite NICE recommendations for home sleep tests,^11^^,^^42^ only 9% of the population was evaluated using this method, indicating underutilization of home testing. In contrast, 29% underwent PSG (a more resource-intensive tool). These disparities between current recommendations and clinical practice may stem from regional variations in diagnostic capabilities and a disconnect between service delivery and budget allocation, where hospitals are not equipped to provide appropriate testing or do not allocate resources aligned with service needs/guideline recommendations. Inefficiencies in diagnostic practices contribute to extended waiting times, such as the median delay of > 6 months from GP referral to CPAP initiation in our study. By enabling patients to undergo testing at home, home testing devices can bridge regional disparities, enhance accessibility, and expedite diagnostic processes. Their adoption in routine clinical practice is essential for aligning with updated guidelines and improving patient pathways. However, undercoding or miscoding for the tools and tests used in the current data set could also have contributed to the low reported usage of home sleep testing.

Key delays were observed between GP referral, OSA diagnosis, and CPAP initiation, with regional variability. Although some regions provided faster appointments and diagnoses, others showed significant delays, especially in the total waiting time to CPAP therapy. This could indicate more efficient processes in some locations, and highlights areas where improvement is needed. Although the recommended treatment pathways differ between countries, an analysis of German health insurance data found that the median time between the first polygraphy and positive airway pressure prescription was 90 days,^36^ similar to the median 70-day delay between OSA diagnosis and starting CPAP in our study. Our data do not provide information about reasons for delays in OSA diagnosis and treatment, but discrepancies between demand and capacity could contribute.

In our study, 42% of patients had no sleep clinic visits after CPAP initiation, contrary to the NICE follow-up protocol.^11^ In Europe, OSA telemonitoring usage grew from 2010 to 2020.^19^ In the United Kingdom, telemonitoring was first recommended in 2021 and therefore likely underused in our sample. Even if telemonitoring was used in some patients, health care contacts should still be coded in the health record. Therefore, lack of formal sleep clinic follow-up in nearly one-half of all patients on CPAP could suggest that opportunities to optimize device usage and prevent therapy termination were lost. Use of telemedicine-based follow-up is an important tool, and has been shown to improve adherence in a meta-analysis.^43^

Strengths of the current study include the use of a large, representative data set as the information source. Both the HES and CPRD are established data sources for research purposes and have been validated for use in epidemiologic research and for submission for health technology assessments. The CPRD includes > 16 million currently registered patients, covering 28% of the UK population. Despite this, there are several limitations that need to be considered when interpreting our findings. First, we used electronic health records data that were entered as part of routine clinical care. Therefore, the information extracted from the databases is dependent on the completeness of patient records and the accuracy of data entry. In addition, missing data were not substituted or imputed, and the impact of any missing data on our study findings (especially for key variables such as OSA diagnoses or follow-up) cannot be determined. The key pieces of data not captured by the databases, and therefore also not by our analyses, relate to OSA severity, symptom burden, and data from overnight sleep studies. This means that we are unable to stratify the OSA group by disease severity, which is an important factor to consider in clinical decision-making about appropriate patient management. We are also unable to determine how diagnostic and treatment approaches vary by OSA severity.

Second, we may only have an incomplete picture of the approaches to OSA diagnosis due to undercoding or miscoding for the tools and tests used. For example, the ESS was used in only 28% of patients overall, and this rate would be expected to be higher. Third, our requirement that individuals included in the study had linked HES and CPRD data resulted in a smaller sample size than would have been possible with the use of the CPRD alone. Nevertheless, the HES contains information that was essential for our analyses. Finally, CPRD-HES linked data are limited to patients registered with GP practices in England only, which could limit the applicability of our findings to other parts of the United Kingdom and the information that the databases contain may not be comprehensive enough to fully represent OSA management in the whole of England (especially in the context of the regional variations documented in our analysis). In addition, the data are specific to the English/UK health system, limiting external generalizability to other health settings and countries.

## Interpretation

This study provided up-to-date real-world evidence on the burden of OSA and CPAP treatment in England in terms of incidence of OSA, patient characteristics, and diagnostic and treatment pathways. The proportion of people with OSA who were treated with CPAP was smaller than the number who did not receive CPAP, and waiting times for diagnosis and treatment were often substantial. Urgent systemic improvements are needed to overcome barriers in OSA management and to optimize clinical pathways, including improved diagnostic and treatment frameworks to expedite care, to ensure timely and effective management of OSA, and to contribute to better long-term health outcomes.

## Funding/Support

This study was funded by 10.13039/100017647ResMed.

## Financial/Nonfinancial Disclosures

The authors have reported to *CHEST Pulmonary* the following: M. D., A. G., and N. A. are employees of ResMed and are ResMed shareholders. F. S. H. and S. B. are employees of OPEN Health. None declared (A. A.).

## Supplementary Data

e-Online Data
