# Supplementary material for: Incidence, Characteristics, and Management Pathways of Obstructive Sleep Apnea in England: A 10-Year Retrospective Cohort Study
Source: CHEST Pulm. 2025 Oct 10;4(1):100221. doi: 10.1016/j.chpulm.2025.100221 (PMC13419131; doi:10.1016/j.chpulm.2025.100221)
Supplement: e-Online Data [file mmc1.pdf]

SUPPLEMENTAL MATERIAL

Incidence, Characteristics, and Management Pathways of Obstructive Sleep Apnea in England: A 10-Year Retrospective Cohort Study

SUPPLEMENTARY METHODS

e-Table 1. Obstructive sleep apnea (OSA) diagnostic codes.

OSA codes	Snomed ct concept id	Medcode	Read code
Obstructive sleep apnea	78275009	8148	Fy03.11
Obstructive sleep apnea	78275009	20748	H5B0.00

e-Table 2. Continuous positive airway pressure (CPAP) codes.

Treatment codes	Snomed ct concept id	Medcode	Read code
CPAP - Continuous positive airway pressure	47545007	22418	8724.00
CPAP - Continuous positive airway pressure	47545007	28444	Z6M1.11
CPAP - Continuous positive airway pressure therapy	47545007	36749	Z6M1.13
Continuous positive airway pressure	47545007	50135	Z6M1.00
Continuous positive airway pressure therapy	47545007	63380	Z6M1.12

e-Table 3. Diagnostic test codes.

Diagnostic test	Code	Source
Respiratory sleep study	DZ50Z	HRG
Polysomnography	U331	OPCS
Overnight oximetry	E913	OPCS
Sleep studies NEC includes polysomnography	A847	OPCS
Sleeping disorders affecting breathing	DZ18Z	HRG
Outpatient respiratory sleep studies	U33.1 + DZ50Z	MIXED

e-Table 4. Epworth Sleepiness Scale (ESS) and Berlin Questionnaire codes

Test	Snomed ct concept id	Medcode
ESS score	763254009	496211000000119
ESS	708735004	763851000006117
ESS	1583951000006100	158395100000611 6
ESS	708735004	11928531000006114
ESS	708735004.00	11928531000006100
ESS score - sitting and reading	1841281000006108	1841281000006112
ESS score - watching television	1841291000006106	1841291000006110
ESS score - sitting inactive in public place	1841301000006107	1841301000006111
ESS score - passenger in car for an hour without break	1841311000006105	1841311000006114
ESS score - lying down to rest in afternoon	1841321000006102	1841321000006118
ESS score - sitting and talking to someone	1841331000006104	1841331000006115
ESS score - sitting quietly after lunch without alcohol	1841341000006109	1841341000006113
ESS score - in a car, stopped for a few mins in traffic	1841351000006106	1841351000006110
ESS score	763254009	85109
Berlin questionnaire for sleep apnea	445483007	100177

SUPPLEMENTARY RESULTS

e-Table 5. Participant characteristics in the sensitivity analysis population, overall and by diagnosis/treatment subgroup

	**Overall (n=75,383)**	**OSA & CPAP (n=15,616)**	**OSA without CPAP (n=17,257)**	**No OSA (n=42,510)**
Age at diagnosis, years	53.5±13.3	54.0±12.8	53.1±13.2	53.4±13.5
Age group, n (%)				
18–20 years	275 (0.36)	35 (0.22)	60 (0.35)	175 (0.41)
21–30 years	3185 (4.23)	525 (3.36)	755 (4.38)	1905 (4.48)
31–40 years	9520 (12.63)	1835 (11.75)	2220 (12.86)	5460 (12.84)
41–50 years	17980 (23.85)	3715 (23.79)	4260 (24.69)	10000 (23.52)
51–60 years	21485 (28.50)	4670 (29.91)	4900 (28.39)	11915 (28.03)
61–70 years	15030 (19.94)	3240 (20.75)	3380 (19.59)	8410 (19.78)
71–80 years	6685 (8.87)	1385 (8.87)	1415 (8.20)	3885 (9.14)
81–90 years	1195 (1.59)	210 (1.34)	260 (1.51)	725 (1.71)
91–100 years	35 (0.05)	<7	<7	30 (0.07)
Male sex, n (%)	52426 (69.55)	10843 (69.44)	12240 (70.93)	29343 (69.03)
Body weight, kg	105.9±25.4	107.6±25.5	106.2±25.5	105.1±25.3
Body mass index, kg/m^2	35.9±8.5	36.5±8.6	35.9±8.6	35.7±8.4
Nonsmoker, n (%)	56228 (74.59)	11785 (75.47)	12626 (73.16)	31817 (74.85)
IMD score (in quintiles)[a]				
≥34.18	16855 (22.00)	3660 (23.00)	3865 (22.00)	9330 (22.00)
21.36–34.17	16265 (22.00)	2875 (18.00)	3675 (21.00)	9380 (22.00)
13.8–21.35	14690 (19.00)	3015 (19.00)	3400 (20.00)	8275 (19.00)
8.50–13.79	14290 (19.00)	3210 (21.00)	3365 (19.00)	8050 (19.00)
≤8.49	13205 (18.00)	2840 (18.00)	2935 (17.00)	7430 (17.00)
Unknown	80 (0.11)	10 (0.06)	20 (0.12)	45 (0.11)
CCI	1.1±1.5	1.2±1.5	1.1±1.4	1.1±1.5
CCI group, n (%)				
0	34652 (45.97)	6845 (43.83)	8149 (47.22)	19562 (46.02)
1–2 (mild)	29530 (39.17)	6252 (40.04)	6695 (38.80)	16662 (39.20)
3–4 (moderate)	8455 (11.22)	1889 (12.10)	1831 (10.61)	4748 (11.17)
5 (severe)	2746 (3.64)	630 (4.03)	582 (3.37)	1538 (3.62)
**Common comorbidities, n (%)**				
Obesity	63314 (83.99)	13617 (87.20)	14504 (84.05)	35193 (82.79)
Cardiovascular disorders	29208 (38.75)	6584 (42.16)	6662 (38.60)	15962 (37.55)
Metabolic disorders	18267 (24.23)	4092 (26.20)	4099 (23.75)	10076 (23.70)
Respiratory disorders	16647 (22.08)	3579 (22.92)	3763 (21.81)	9305 (21.89)
Psychiatric disorders	12682 (16.82)	2700 (17.29)	2743 (15.89)	7239 (17.03)

Values are mean ± standard deviation, or number of participants (%).

[a]Higher scores indicate greater deprivation.

CCI, Charlson Comorbidity Index; CPAP, continuous positive airway pressure; IMD, Index of Multiple Deprivation; OSA, obstructive sleep apnea.

e-Table 6. Diagnostic tests in the overall sensitivity analysis population, in age subgroups and in subgroups based on diagnosis/treatment

Number of participants (%)	Overall (n=75,883)	Age <50 years (n=28,792)	Age 50–69 years (n=37,537)	Age ≥70 years (n=9,054)
**By age group**				
Berlin questionnaire	197 (0.26)	81 (0.28)	104 (0.28)	12 (0.13)
Epworth Sleepiness Scale	21,627 (28.69)	8,835 (30.69)	10,770 (28.69)	2,022 (22.33)
Home sleep study	6,890 (9.14)	2,731 (9.49)	3,477 (9.26)	682 (7.53)
Pulse oximetry	8,711 (11.56)	2,946 (10.23)	4,459 (11.88)	1,306 (14.42)
PSG	21,566 (28.61)	8,103 (28.14)	10,935 (29.13)	2,528 (27.92)
Pulse oximetry only	6,767 (8.98)	2,372 (8.24)	3,416 (9.10)	979 (10.81)
Home sleep study only	<7	0 (0.00)	<7	<7
PSG only	13,055 (17.32)	4,903 (17.03)	6,585 (17.54)	1,567 (17.31)
Pulse oximetry + home sleep study	<7	<7	<7	0 (0.00)
Pulse oximetry + home sleep study + PSG	312 (0.41)	103 (0.36)	163 (0.43)	46 (0.51)
Home sleep study + PSG	6,569 (8.71)	2,627 (9.12)	3,308 (8.81)	634 (7.00)
Pulse oximetry + PSG	1,630 (2.16)	470 (1.63)	879 (2.34)	281 (3.10)
		**OSA & CPAP (n=15,616)**	**OSA without CPAP (n=17,257)**	**No OSA (n=42,510)**
**By diagnosis/treatment**				
Berlin questionnaire	-	53 (0.34)	39 (0.23)	105 (0.25)
Epworth Sleepiness Scale	-	5,436 (34.81)	4,857 (28.15)	11,334 (26.66)
Home sleep study	-	1,662 (10.64)	1,524 (8.83)	3,704 (8.71)
Pulse oximetry	-	1,684 (10.78)	1,655 (9.59)	5,372 (12.64)
PSG	-	4,730 (30.29)	4,401 (25.50)	12,435 (29.25)
Pulse oximetry only	-	1,310 (8.39)	1,303 (7.55)	4,154 (9.77)
Home sleep study only	-	<7	<7	<7
PSG only	-	2,762 (17.69)	2,591 (15.01)	7,702 (18.12)
Pulse oximetry + home sleep study	-	0 (0.00)	<7	<7
Pulse oximetry + home sleep study + PSG	-	66 (0.42)	61 (0.35)	185 (0.44)
Home sleep study + PSG	-	1,594 (10.21)	1,459 (8.45)	3,516 (8.27)
Pulse oximetry + PSG	-	308 (1.97)	290 (1.68)	1,032 (2.43)

Values are number of participants (%).
CPAP, continuous positive airway pressure; OSA, obstructive sleep apnea; PSG, polysomnography.

e-Table 7. Waiting times in the sensitivity analysis population, in age subgroups and in subgroups based on diagnosis/treatment

Median (IQR) wait times, days	Overall (n=75,883)	Age <50 years (n=28,792)	Age 50–69 years (n=37,537)	Age ≥70 years (n=9,054)
**By age group**				
For OSA appointment	37 (20; 64)	39 (21; 67)	36 (19; 63)	35 (16; 61)
Initial sleep clinic visit to first diagnosis of OSA	52 (11; 159)	48 (8; 134)	54 (12; 166)	62 (14; 244)
OSA diagnosis to CPAP initiation	64 (0; 411)	68 (0; 412)	64 (0; 420)	58 (0; 374)
Total time until CPAP initiation	194 (90; 799)	187 (89; 680)	195 (91; 841)	213 (88; 831)
**By diagnosis/treatment**		**OSA & CPAP (n=15,616)**	**OSA without CPAP (n=17,257)**	**No OSA (n=42,510)**
For OSA appointment	-	36 (20; 61)	38 (19; 64)	38 (20; 66)
Initial sleep clinic visit to first diagnosis of OSA	-	49 (9; 144)	45 (6; 134)	N/A
OSA diagnosis to CPAP initiation	-	64 (0; 411)	N/A	N/A
Total time until CPAP initiation	-	194 (90; 799)	N/A	N/A

Values are number of participants (%) or median (interquartile range).
CPAP, continuous positive airway pressure; IQR, interquartile range; N/A, not applicable; OSA, obstructive sleep apnea.
